# Electrospun Eudragit Nanofiber Mats as a Buccal Delivery Platform for Triamcinolone Acetonide

**DOI:** 10.1021/acsomega.6c02535

**Published:** 2026-09-14

**Authors:** Nilay Doğan, Muhammet Davut Arpa, Evrim Akyıl

**Affiliations:** † Istanbul Medipol University, School of Pharmacy, Department of Pharmaceutical Technology, 34815, Istanbul, Turkey; ‡ Institute of Graduate Education, Department of Pharmaceutical Technology, 52944 Anadolu University, 26470, Eskişehir, Turkey; § Anadolu University, Faculty of Pharmacy, Department of Pharmaceutical Technology, 26470, Eskişehir, Turkey

## Abstract

Recurrent aphthous stomatitis is the most frequently observed disorder of the oral mucosa. Since the etiopathogenesis of recurrent aphthous stomatitis is unclear, there is no specific treatment method. The preferred treatment methods aim to alleviate symptoms such as pain and discomfort and shorten the recovery period. Triamcinolone acetonide is one of the most commonly used topical glucocorticoids. Within the scope of this study, electrospun nanofiber-based formulations were developed using two Eudragit (E) polymers, S100 and RL100, as an alternative to the marketed product, which contains 0.1% triamcinolone acetonide (Kenacort-A orobase ointment). Drug loading capacities of the nanofiber films (per 4 cm^2^) were 1.333 ± 0.594 mg for E S and 1.969 ± 1.000 mg for E RL, while SEM analysis demonstrated that the average fiber diameters were 259 ± 83 nm and 192 ± 81 nm for E S and E RL, respectively. FTIR, NMR, and DSC analyses confirmed the successful incorporation of triamcinolone acetonide into the nanofiber matrix and indicated the absence of any chemical incompatibility between the drug and the polymers. Drug release studies and permeability studies demonstrated higher drug release and permeation levels compared to the commercial product, with an improved release profile achieved by the developed nanofiber formulations. As a result, triamcinolone acetonide-loaded nanofibers were successfully produced and characterized, and the results indicate that these nanofiber formulations represent a promising alternative to existing topical treatments for recurrent aphthous stomatitis.

## Graphical abstract

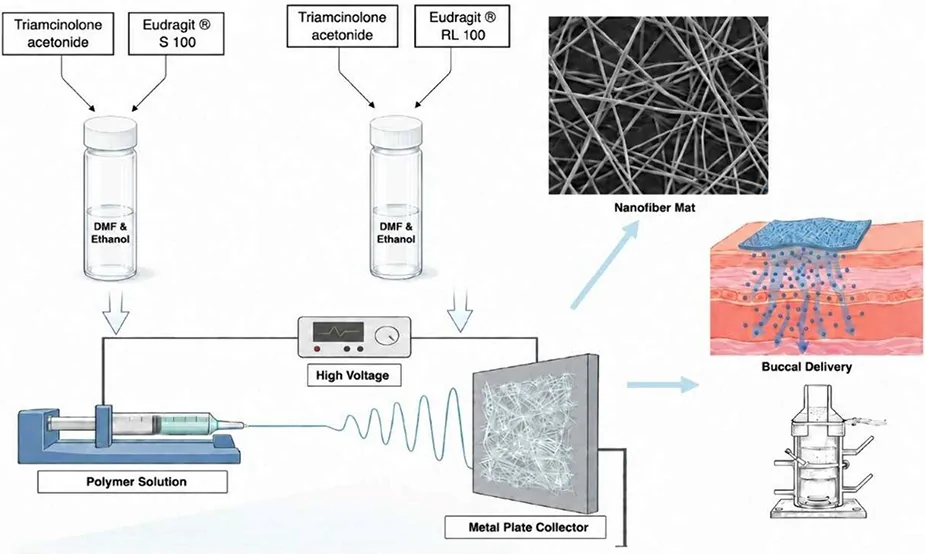

## 1 Introduction

Ulceration is a common clinical disorder observed in various oral diseases and may arise from a wide range of etiological factors. These oral conditions may originate primarily within the oral cavity or may develop secondarily as complications of underlying systemic disorders. Among primary conditions, recurrent aphthous stomatitis (RAS), also termed canker sores, is the most prevalent inflammatory condition of the oral cavity.^1,2^ Although its exact pathogenesis remains unclear, RAS is considered to be multifactorial in nature, involving a multifactorial interplay of genetic predisposition with various exogenous and endogenous triggers, including microbial infections (viral or bacterial), dietary components, hormones, psychological stress, pharmacological agents, nutritional deficiencies, and systemic conditions.^3,4^ Since the etiopathogenesis of RAS remains unclear, there is no specific treatment. The preferred treatment methods aim to control the symptoms (pain and discomfort) and shorten the recovery period. Topical corticosteroids are the most commonly used medication for treating RAS.^5^ They represent the first-line treatment approach due to their efficacy in reducing inflammation, alleviating pain, and shortening the duration of ulcerative lesions. By minimizing discomfort, these agents facilitate routine oral functions such as eating, speaking, and performing oral hygiene. Topical glucocorticoids are particularly effective in the active phase of the disease and are usually applied multiple times daily.^6^

Triamcinolone acetonide (TA) is a synthetic glucocorticoid characterized by a prolonged duration of action and greater anti-inflammatory potency compared to equivalent doses of prednisone.^7,8^ It is also the most commonly used topical glucocorticoid for the treatment of recurrent aphthous stomatitis (RAS). It is typically applied three to ten times per day at concentrations ranging from 0.05% to 0.5%, with 0.1% being the most frequently used and widely supported concentration. This dosage has been reported as the most effective concentration for managing RAS symptoms.^9^ Although conventional preparations such as ointments, creams, and gels are widely used for the local treatment and provide high drug concentrations at the site of application, they are generally considered suboptimal due to their rapid clearance from the mucosal surface. This is primarily attributed to the physiological cleansing mechanisms of the oral cavity, including continuous salivary flow, swallowing, tongue movements, chewing, and speech. These factors reduce the contact time between the drug and the mucosa, leading to inconsistent drug distribution and a decline in the therapeutic drug concentration at the target site.^10–12^ For this reason, innovative therapeutic strategies are required to overcome the limitations of conventional treatments and to improve the therapeutic effect. Owing to these limitations, nanofibers have gained considerable attention in biomedical and pharmaceutical research. Nanofibers are systems with high flexibility and nanometer diameters produced from various sources, such as natural or synthetic polymers, composite structures, inorganic nanomaterials, and biomolecules. In addition to their flexibility, nanofibers also have a high surface area-to-volume ratio.^13,14^ Due to these characteristics, nanofibers are employed in a broad range of applications, including filtration, the aerospace industry, tissue engineering, energy production, information technologies, protective clothing production, optical applications, and nanosensor development.^15,16^ Beyond these applications, nanofibers are also used in the development of drug delivery systems by increasing the dissolution rate and solubility of poorly absorbed drugs, improving their bioavailability and release properties.^17^ Buccal application of nanofibers typically enables the dissolution of drug-loaded electrospun mats upon contact with the mucosal surface, facilitating the direct absorption of the active compound into the submucosal capillary network.^18^ The nanofiber patch adheres to the ulcer site and releases the drug. Therefore, the polymers used in oral ulcer treatment should exhibit good biocompatibility and bioadhesive properties.^19^ Various techniques can be employed for the production of nanofibers. Electrospinning refers to the process by which a polymeric solution or melt is subjected to an electric field, resulting in the formation of nanofibers.^20^ This technique enables the formation of nanofibers by applying an electric field between the nozzle and the collector, causing the charged polymer solution to form a Taylor cone. As the electrostatic forces overcome surface tension, the solution is ejected, and the solvent rapidly evaporates during flight, resulting in solid nanofibers deposited on the collector.^21^ In addition to conventional electrospinning, electrohydrodynamic atomization (EHDA) techniques, including electrospraying, have attracted increasing attention as versatile platforms for the fabrication of functional micro and nanostructured materials. These techniques enable the processing of a wide range of polymers and active pharmaceutical ingredients into tailored delivery systems with controlled morphology and performance. Over the past decade, electrospinning has evolved from simple single-fluid systems to more advanced configurations such as coaxial, side-by-side, triaxial, and multifluid electrospinning approaches, which allow for the precise engineering of core–shell or multilayered nanostructures and improved control over drug loading and release kinetics.^22–24^ This evolution has significantly expanded the process–structure–performance relationships achievable with electrospun systems. In contrast, electrospraying offers complementary advantages by producing micro- or nanoparticles with tunable size distribution and surface properties.^25,26^ Among the polymers used in electrospinning, Eudragit is one of the most versatile materials due to its physicochemical and release-controlling properties. Eudragit is the commercial name for methacrylic acid copolymers, whose properties vary depending on their substituents. These polymers are widely used in pharmaceutical formulations for purposes such as enteric coating, taste masking, sustained release, and moisture protection.^27^ Eudragit polymers have been used as electrospinnable carriers in buccal nanofiber formulations due to their solubility and bioadhesive properties.^28,29^ Despite these advances, the application of Eudragit polymers within such advanced electrohydrodynamic systems remains relatively limited, particularly in the context of buccal drug delivery.^30,31^ This represents an important opportunity for further exploration, as the integration of Eudragit polymers with electrospinning techniques may provide novel platforms with enhanced functional performance for localized drug delivery applications. Therefore, the present study aims to contribute to this limited area by developing Eudragit-based electrospun nanofibers for buccal drug delivery.^27–29^ Eudragit S100 and Eudragit RL100 were selected because they represent two structurally and functionally distinct methacrylic acid copolymers within the Eudragit family. Eudragit S100 is an anionic pH-dependent polymer, while Eudragit RL100 contains quaternary ammonium groups and is characterized by higher permeability.^32,33^ Accordingly, comparison of these two polymers enabled the evaluation of how polymer ionicity affects electrospinnability and the parameters, drug incorporation, release behavior, and buccal permeation performance.

Previous studies have reported the development of triamcinolone-loaded nanofibers and nanostructures for various biomedical applications. Specifically, for buccal delivery,^34^ developed gliadin- and ethylcellulose-based electrospun fibrous mats containing triamcinolone base, demonstrating promising results in terms of biocompatibility and anti-inflammatory effects for the treatment of oral ulcers. In another study, Payab et al., (2016) prepared TA-loaded nanofiber and nanobead structures using Eudragit RS100 and investigated their morphological and physicochemical properties without specifying a particular route of administration. In addition, TA-containing nanofiber systems have been reported for applications outside of buccal use; for example, chitosan-based electrospun nanofibers were prepared and compared with hydrophobic polymer-based nanofibers containing Eudragit S100 and zein as potential ocular inserts.^36^ These studies demonstrate that triamcinolone base and TA can be successfully integrated into nanofiber-based carrier systems, while also indicating areas for improvement, particularly in polymer selection and formulation strategies for buccal applications. Additionally, to the best of our knowledge, this study represents one of the first *ex vivo* permeation comparisons with a marketed TA buccal formulation using Franz diffusion cells, highlighting the potential of nanofiber-based systems as an alternative platform for buccal drug delivery.

This study aimed to develop a nanofiber-based system for localized buccal application of TA in the treatment of oral lesions as an alternative to traditional semisolid preparations containing TA. To this end, electrospun nanofibers were developed using two different Eudragit polymers in distinct formulation systems and comparatively evaluated and subsequently characterized. *In vitro* release and *ex vivo* permeability studies were conducted for the lead formulations.

## 2 Materials and Method

### 2.1 Materials

TA was kindly gifted by DEVA Holding (Türkiye). Eudragit S100 and Eudragit RL100 were purchased from Evonik (Germany). N,N-dimethylformamide (DMF) and deuterated chloroform were obtained from Merck (Germany). Methanol and ethanol were obtained from Carlo Erba (Germany). Phosphate-buffered tablets (pH 6.8 and pH 7.4) were obtained from Sigma-Aldrich (USA).

### 2.2 Development of Analytical Method and Validation

Throughout the study, an analytical method compatible with the physicochemical properties of TA was developed to enable its quantitative determination. An HPLC system (Agilent 1100 Series, USA) was used with a C18 column (4.6 × 150 mm, 5 μm, AISIMO; China), the mobile phase consisted of distilled water and methanol (30:70; v/v), with a flow rate of 1.0 mL/min. The sample injection volume was set at 20 μL, and the column temperature was maintained at 25 °C.^37^ Maximum wavelength was detected as 238 nm using a UV–vis spectrophotometer (UV-1800, Shimadzu, Japan). The analytical method developed for the quantification of TA was validated in accordance with the ICH Q2­(R2) guideline.^38^

### 2.3 Solubility Studies

The studies were carried out in microcentrifuge tubes with three replicates for each solvent. Initially, 1 mg of the TA was added to 1 mL of solvent and vortexed to dissolve. Additional TA was incrementally added and vortexed until turbidity was observed. The microcentrifuge tubes showing turbidity were tightly sealed with parafilm, placed in a shaker (SSL2, Stuart, UK), and shaken at 250 rpm at room temperature for 24 h. After the period, the samples were centrifuged (3–18 KS, Sigma, Germany) at 13,200 rpm for 15 min, and a 0.5 mL aliquot of the clear supernatant was analyzed by HPLC.^39^

### 2.4 Electrospinning Process

The electrospinning process is influenced by many parameters, which can be classified into three main groups: processing factors (e.g., flow rate, voltage, and needle-to-collector distance), solution-related factors (e.g., concentration and viscosity), and ambient factors (e.g., temperature and humidity). Each of these factors plays a critical role in fiber morphology, continuity, and overall nanofiber formation efficiency.^22^ Process parameters were determined based on the formation of a stable and continuous jet, the presence of a Taylor cone, and the absence of freezing or clogging at the nozzle tip. Based on these parameters and criteria, the same production conditions were successfully provided in the device for both polymers. In this study, the electrospinning process was performed using a Nanospinner system (NS Plus, Inovenso, USA). These processing conditions were further supported by SEM-based morphological evaluation, which confirmed bead-free nanofiber formation for the optimized formulations. For the process, a metal plate was used as the collector. The surface of the metal plate was covered with aluminum foil, the electrospun nanofibers were collected onto the foil, and these parameters are as follows: 15 cm was the nozzle-to-collector distance, 1 mL/h was the flow rate, and 16 kV was the applied voltage. A 15-gauge metal nozzle was used, and the collector movement speed was set to 5 mm/s.

#### 2.4.1 Preparation and Evaluation of Blank Nanofiber Formulations

The target was to obtain nanofiber formulations with uniform morphology without bead formation and a successful electrospinning process. To determine the optimum formulations, polymer solutions were first prepared, followed by the electrospinning process. For solution preparation, accurately weighed polymers were placed in 40 mL screw-capped glass vials, and the required solvent mixture was added. The mixtures were stirred using a magnetic stirrer (RT 10, IKA, Germany) for 24 h at room temperature. The prepared polymer solutions were electrospun to obtain nanofiber mats. The morphological properties were evaluated using scanning electron microscopy (SEM-TM3030 Plus, Hitachi, Japan). The contents of the blank nanofiber formulations are listed in Tables 1 and 2 below.

**Table 1:** Blank Nanofiber Formulations Containing Eudragit S100 (E S)

Formulation Code	Ethanol (mL)	DMF (mL)	Polymer Amount (g)
E S 10	5	5	1.00
E S 12.5	5	5	1.25
E S 15	5	5	1.50
E S 17.5	5	5	1.75
E S 20	5	5	2.00
E S 22.5	5	5	2.25
E S 25	5	5	2.50
E S 27.5	5	5	2.75
E S 30	5	5	3.00

**Table 2:** Blank Nanofiber Formulations Containing Eudragit RL100 (E RL)

Formulation Code	Ethanol (mL)	DMF (mL)	Polymer Amount (g)
E RL 10	7	3	1.00
E RL 12.5	7	3	1.25
E RL 15	7	3	1.50
E RL 17.5	7	3	1.75
E RL 20	7	3	2.00
E RL 22.5	7	3	2.25
E RL 25	7	3	2.50
E RL 27.5	7	3	2.75
E RL 30	7	3	3.00
E RL 32.5	7	3	3.25
E RL 35	7	3	3.50

#### 2.4.2 Preparation of TA-Loaded Nanofiber Formulations

Based on the evaluation of the blank nanofibers presented in Section 2.4.1, the selected nanofiber formulations were loaded with TA at a concentration corresponding to 20% of the polymer content. The polymer and TA were dissolved in the previously described solvent system as detailed in Section 2.4.1, prior the electrospinning. The solutions were stirred at the speeds specified in Tables 1 and 2 using the magnetic stirrer. The morphological evaluation of these TA-loaded nanofiber mats were performed using SEM. The mechanical properties of the TA-loaded nanofiber mats were assessed, and the studies on content uniformity were conducted. According to the results, optimum formulations were chosen for the following drug release and permeability studies.

#### 2.4.3 Characterization of TA-Loaded Nanofiber Formulations

##### 2.4.3.1 Mechanical Properties of TA-Loaded Nanofiber Formulations

To determine the mechanical properties of the formulations, texture profile analysis was conducted using a texture analyzer (TA.XT*plus* C, Stable Micro Systems, United Kingdom). The samples were cut into 3 × 2 cm strips. Each sample was fixed vertically to the tension probe by taping 0.5 cm from both the top and bottom ends, with the 3 cm side positioned perpendicular to the direction of the applied force. The test was initiated at a speed of 0.5 mm/s, and the force was recorded by the device software. The key parameters, including elongation at break (%) and tensile strength, were determined. For each formulation, tests were conducted in triplicate.

##### 2.4.3.2 Content Uniformity of TA-Loaded Nanofiber Formulations

In content uniformity studies, the amount of TA in the TA-loaded nanofiber mat formulations was determined considering the intended site of application. Accordingly, the nanofiber mats were cut into 4 cm^2^ pieces by slicing into 2 cm-wide vertical and horizontal strips. These pieces were diluted with the mobile phase and analyzed. The amount of TA per 4 cm^2^ was quantified by adjusting the movement stroke setting of the electrospinning device.

##### 2.4.3.3 Morphological Characterization of Optimum Formulations

To determine fiber diameter, SEM images of the optimized formulations were obtained at 10k × magnification. The diameters of 15 different fibers were measured at this magnification, and the data were reported as mean ± SD.

##### 2.4.3.4 Thermal Analyses

Thermal analyses of TA, the polymers, and the blank and loaded optimal nanofibers were performed using a differential scanning calorimetry (DSC-60, Shimadzu, Japan). Each sample (∼5 mg) was weighed into sealed aluminum pans and was analyzed over a temperature range of 30–300 °C under a nitrogen atmosphere (50 mL/min) at a heating rate of 10 °C/min.^40^

##### 2.4.3.5 Spectroscopic Analyses

The infrared spectra of TA, the polymers, and the blank and loaded optimal formulations were performed using Fourier-transform infrared (FTIR) spectroscopy (UATR Two, PerkinElmer, USA) over a wavelength range of 400–4000 cm^–1^, to evaluate potential chemical interactions and characterize the functional groups within the components.^41^ Additionally, ^1^H NMR spectroscopy was employed to further confirm the chemical structure and interactions within the formulations (UltraShield, Bruker, Germany). The analysis used deuterated chloroform as the solvent.^42^

### 2.5 *In Vitro* Drug Release Studies

Drug release studies of the nanofiber mats were carried out using a Franz diffusion cell (receptor volume: 12 mL, diffusion area: 1.77 cm^2^) system with 6 cells at 37 ± 2 °C.^41^ The receptor compartment had a volume of. Based on the diffusion area, nanofiber samples were cut into 0.5 × 0.5 cm^2^ pieces. The commercial product (Kenacort Orabase ointment, 0.1% TA, Deva Holding, Türkiye) was used as the control group. The amount of ointment used was calculated based on the average drug content determined in the content uniformity studies. Drug release experiments for the formulations and the commercial product were performed in quadruplicate.

To ensure sink conditions, the release medium was composed of a phosphate-buffered solution (pH 7.4) and ethanol (60:40, v/v) and was continuously stirred at 300 rpm throughout the experiment. A cellulose dialysis membrane (12–14,000 kDa, Sigma-Aldrich, USA) was placed between the receptor and donor compartments, and the membrane surface was prewetted with 100 μL of phosphate-buffered solution (pH 6.8). After a 30 min conditioning period, the formulations were applied, and the release study was initiated. At predetermined intervals (5, 15, 30, 45, 60, and 90 min; 2, 4, 6, 8, 10, 12, and 24 h), the samples (0.5 mL) were collected and immediately replaced with fresh medium to maintain a constant receptor volume. The collected samples were filtered through a cellulose acetate membrane filter (0.22 μm, Labmarker, Türkiye) and analyzed. In addition, the release profiles were evaluated to determine the release kinetics of the nanofiber formulations and the commercial product.^43^

### 2.6 *Ex Vivo* Permeability Studies

The permeability studies were performed using the same Franz diffusion cell system and experimental conditions described in the *in vitro* release studies, with all tests conducted in quadruplicate. Sink conditions, receptor medium composition, stirring rate, sampling intervals, and sample filtration procedures had remained similar to those performed in the drug release experiments. For these studies, freshly removed bovine buccal mucosa was mounted between the donor and receptor compartments. The tissue was carefully cleaned to remove muscle, nerve, and connective layers, after which the mucosal surface was prewetted with 100 μL of phosphate-buffered solution (pH 6.8) and equilibrated for 30 min before sample application. Following equilibration, the formulations were placed onto the mucosal surface, and the permeation study was initiated. The permeation study was conducted to evaluate drug transport across the superficial layers of buccal tissue and does not imply systemic delivery.

### 2.7 Statistical Analysis

Data are presented as mean ± standard deviation (SD). Statistical analyses were carried out using Microsoft Excel (Microsoft Corporation, Redmond, WA, USA). Differences between the optimized Eudragit S100 and Eudragit RL100 formulations were evaluated using an unpaired Student’s *t* test. A p-value of <0.05 was considered statistically significant.

## 3 Results and Discussion

### 3.1 Development of HPLC Method and Validation

The linearity of the method was evaluated over the concentration range of 2.5–30 μg/mL. Although a minimum of five different concentrations are generally recommended for linearity assessment, ten different concentrations were employed in this study. The calibration curve exhibited excellent linearity with a determination coefficient (r^2^) of 0.9998, described by the regression equation y = 42.5820.x – 7.0215. The LOD and LOQ were determined to be 0.060 ± 0.013 μg/mL and 0.181 ± 0.040 μg/mL, respectively. These low LOD and LOQ values demonstrate the high sensitivity of the developed method for the accurate quantification of TA at low concentrations.

### 3.2 Solubility Studies

The solubility of TA was evaluated in DMF and ethanol, which were used as formulation solvents, as well as in methanol and distilled water, the solvents used in the HPLC mobile phase. In addition, solubility studies were conducted in phosphate buffer solutions at pH 6.8 and pH 7.4 to simulate the physiological pH conditions of the buccal mucosa and blood, respectively. TA showed the highest solubility in DMF with a value of 254 mg/mL. This was followed by methanol (2.59 mg/mL) and ethanol (1.16 mg/mL). This significantly higher solubility in DMF explains its selection as a primary solvent. In contrast, the solubility of TA was considerably lower in aqueous media. Specifically, the solubility in distilled water was 0.0142 mg/mL, in the pH 6.8 buffer was 0.0147 mg/mL, and in the pH 7.4 buffer was 0.0155 mg/mL. Due to the low solubility of TA in water, DMF and ethanol were selected as the solvents in the formulation studies. Since DMF/ethanol was used as the electrospinning solvent system, future studies should also include residual solvent analysis using a validated and solvent-specific analytical method, such as headspace gas chromatography, to confirm the complete removal or acceptable residual levels of organic solvents in the final nanofiber mats prior to buccal application.^44,45^

### 3.3 Electrospinning Process

#### 3.3.1 Preparation and Evaluation of Blank Nanofiber Formulations

Solutions prepared for the blank nanofiber formulations were observed throughout the preparation process. The electrospinning process was monitored, and morphological analyses of samples exhibiting fiber formation were performed using SEM. All observations are presented in Tables 3 and 4, while SEM images are shown in Figure 1. Based on these evaluations, the formulations selected for TA loading were determined as E S 20 for Eudragit S100 and E RL 32.5 for Eudragit RL100. Although the primary aim of this study was not a systematic solvent optimization, the selection of DMF/ethanol mixtures was based on their complementary physicochemical properties. DMF, with its high polarity and strong solvent capacity, ensures effective dissolution of both the polymer and the poorly water-soluble drug, while ethanol, as a more volatile solvent, facilitates rapid solvent evaporation during jet formation. The ratio of these solvents influences key parameters such as solution viscosity, surface tension, and evaporation rate, which in turn govern fiber formation and morphology. At appropriate ratios, a stable Taylor cone and continuous jet formation were achieved, leading to uniform and bead-free nanofibers. In contrast, deviations from this balance resulted in bead formation or process instability, likely due to insufficient chain entanglement or nonuniform solvent evaporation. These observations are consistent with previous reports highlighting the critical role of solvent systems in electrospinning performance.^46,47^

**Figure 1:**
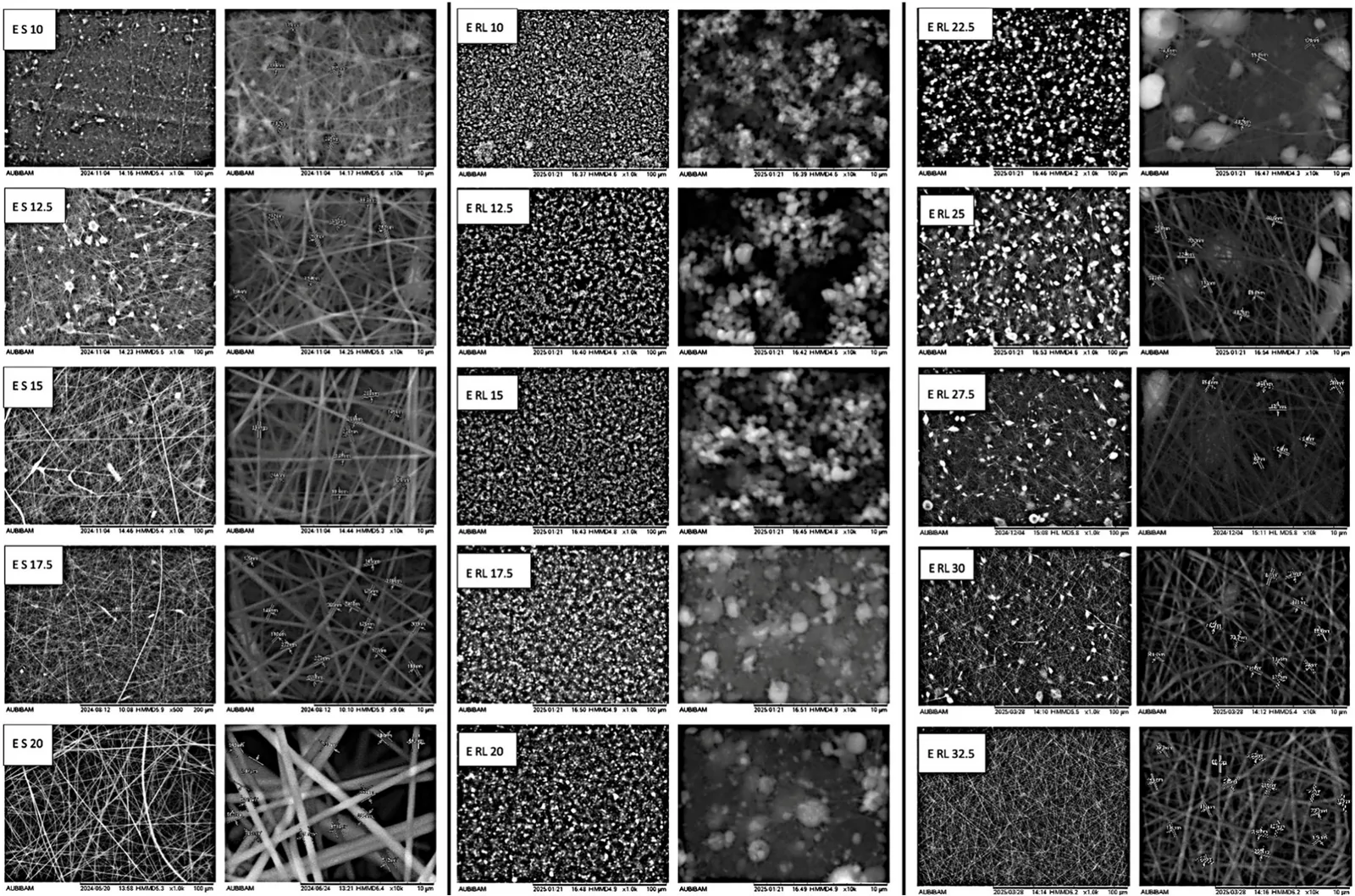
SEM images of the Eudragit S100- and Eudragit RL100-based blank nanofiber formulations.

**Table 3:** Evaluation of Eudragit S100-Based Nanofibers

Formulation Code	Visual evaluation of the solution	Evaluation and characterization of the electrospinning process	Morphological evaluation using SEM^a^
E S 10	Homogeneous	Fiber formation was observed on the collector plate; however, the jet formation was not continuous	+
E S 12.5	Homogeneous	Fiber formation was observed on the collector plate; however, the jet formation was not continuous	+
E S 15	Homogeneous	Continuous and stable jet formation was observed, with fiber formation on the collector plate	+
E S 17.5	Homogeneous	Continuous and stable jet formation was observed, with fiber formation on the collector plate	+
E S 20	Homogeneous	Continuous and stable jet formation was observed, with fiber formation on the collector plate	–
E S 22.5	Homogeneous	Clogging/freezing occurred at the needle tip	SEM image not available
E S 25	Homogeneous	Clogging/freezing occurred at the needle tip	SEM image not available
E S 27.5	TA/polymer solubility problem	Electrospinning could not be performed due to solubility problem	SEM image not available
E S 30	TA/polymer solubility problem	Electrospinning could not be performed due to solubility problem	SEM image not available

a (+: presence of beads, −: bead-free fiber morphology).

**Table 4:** Evaluation of Eudragit RL100-Based Nanofibers

Formulation Code	Visual evaluation of the solution	Evaluation and characterization of the electrospinning process	Morphological evaluation using SEM^a^
E RL 10	Homogeneous	Fiber formation was observed on the collector plate; however, the jet formation was not continuous	+
E RL 12.5	Homogeneous	Fiber formation was observed on the collector plate; however, the jet formation was not continuous	+
E RL 15	Homogeneous	Fiber formation was observed on the collector plate; however, the jet formation was not continuous	+
E RL 17.5	Homogeneous	Fiber formation was observed on the collector plate; however, the jet formation was not continuous	+
E RL 20	Homogeneous	Fiber formation was observed on the collector plate; however, the jet formation was not continuous	+
E RL 22.5	Homogeneous	Fiber formation was observed on the collector plate; however, the jet formation was not continuous	+
E RL 25	Homogeneous	Continuous and stable jet formation was observed, with fiber deposition on the collector plate	+
E RL 27.5	Homogeneous	Continuous and stable jet formation was observed, with fiber deposition on the collector plate	+
E RL 30	Homogeneous	Continuous and stable jet formation was observed, with fiber deposition on the collector plate	+
E RL 32.5	Homogeneous	Continuous and stable jet formation was observed, with fiber deposition on the collector plate	–
E RL 35	Homogeneous	Clogging/freezing occurred at the needle tip	SEM image not available

a (+: presence of beads, −: bead-free fiber morphology).

#### 3.3.2 Characterization of TA-Loaded Nanofiber Formulations

##### 3.3.2.1 Mechanical Properties of TA-Loaded Nanofiber Formulations

The mechanical behavior of both formulations, including tensile strength and elongation behavior, is illustrated in Figure 2. Nanofibers obtained at a 60 mm movement stroke were observed to stretch before separation during analysis. As shown in Table 5, formulations prepared with a 60 mm movement stroke exhibited the highest tensile strength and elongation for both formulations. While the mechanical properties provide preliminary information on the structural integrity of the nanofiber mats, there is no universally accepted tensile strength range for buccal nanofiber formulations because reported values vary depending on polymer composition, solvent content, thickness, preparation method, and testing conditions. In a study, a fabric geometry model was developed to describe the tensile properties of nanofiber mats by analyzing the unit cell structure of an idealized nanofiber and the geometry of a specimen extracted from this unit cell.^48^ According to this model, the width-to-length ratio, the specimen’s porosity, and the inherent tensile characteristics of individual nanofibers all have a major impact on the tensile strength and elastic nature of a nanofiber mat with homogeneous fiber distribution and orientation. It was reported that nanofibers with higher porosity and smaller diameters exhibit lower tensile strength. In line with these findings, the present study also demonstrated that the Eudragit S100 formulation, which exhibited a larger average fiber diameter, had higher tensile strength compared with the Eudragit RL100 formulation, as shown in Table 5.

**Figure 2:**
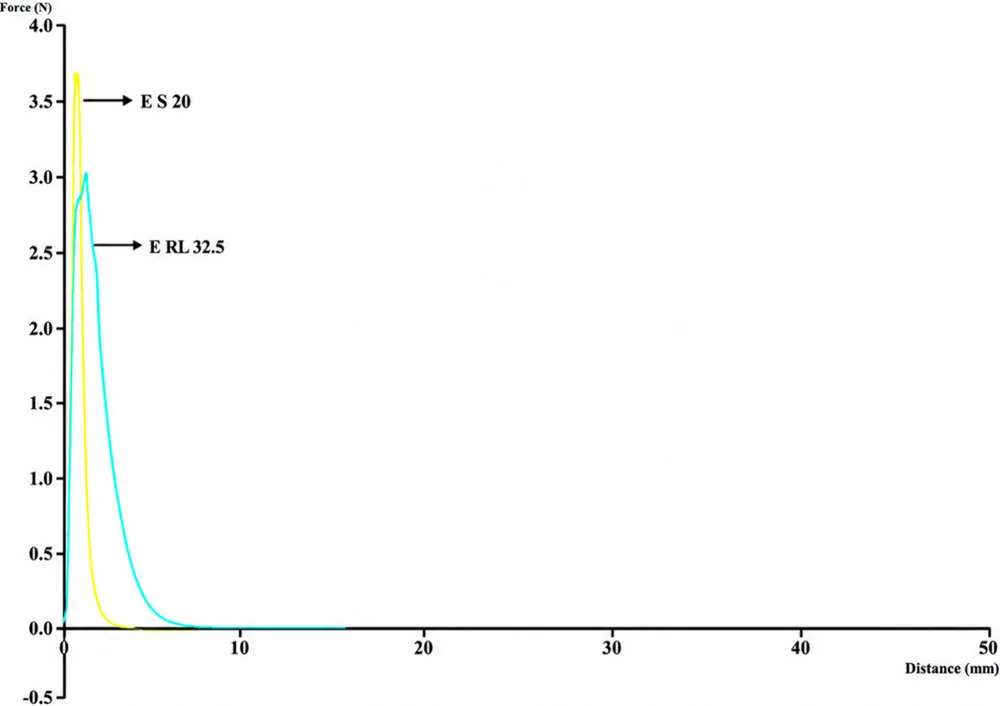
Tensile profiles of the drug-loaded optimum nanofibers.

**Table 5:** Results of the Mechanical Properties of the Drug-Loaded Optimum Nanofibers

Formulation Code	Tensile strength (N/cm^2^)	Elongation at break (%)
E S 20	0.936 ± 0.377	3.055 ± 0.564
E RL 32.5	0.856 ± 0.353	4.459 ± 2.359

##### 3.3.2.2 Content Uniformity of TA-Loaded Nanofiber Formulations

For ideal formulations, the TA amount per 4 cm^2^ was found as 1.333 ± 0.594 mg for E S 20 and 1.969 ± 1.000 mg for E RL 32.5. The amount of TA in the E RL 100-based formulation was higher than that in the E S 100-based formulation. The higher amount of TA measured per 4 cm^2^ in the Eudragit RL100-based formulation (E RL 32.5) compared to the Eudragit S100-based formulation (E S 20) can be attributed to the different physicochemical properties of the two polymers and the differences in fiber morphology formed during electrospinning. The more hydrophobic and permeable structure of Eudragit RL100 may have allowed for more effective retention of TA within the polymer matrix in the electrospinning solution and greater drug loading into the fiber structure. In contrast, the more homogeneous but finer structure of Eudragit S100-based fibers may have resulted in a relatively lower amount of TA retained per unit area. The relatively high standard deviation observed in both formulations stems from the structural nature of the nanofiber mats. Electrospun nanofibers can exhibit a heterogeneous structure in terms of fiber diameter, porosity, layering degree, and local drug distribution. These microstructural variations can cause the amount of TA to vary in 4 cm^2^ sections taken from different sampling regions. In addition, mechanical deformations that may occur during the cutting and handling of nanofiber mats may also have increased the variation in the measured amount of drug.

The relatively high standard deviation observed in drug content per unit area indicates a degree of heterogeneity within the electrospun nanofiber mats. This variability may arise from several factors, including nonuniform fiber deposition during electrospinning, heterogeneous drug distribution within the polymer matrix, and variations introduced during manual cutting and sample handling. Similar challenges have been reported in electrospun drug delivery systems, particularly at the laboratory scale, where process parameters are not yet fully optimized.^16,49^ The intrinsic properties of nanofiber mats, such as high porosity and random fiber orientation, may further contribute to local variations in drug loading.^16,49,50^ From a translational perspective, such variability may pose challenges for dose reproducibility and regulatory acceptance of the final dosage form. However, it is well established that content uniformity in electrospun systems can be significantly improved through process optimization, including controlled fiber collection, automation of cutting processes and scale-up using advanced electrospinning techniques.^51,52^ Therefore, while the present findings reflect early stage formulation characteristics, further optimization would be required to meet the stringent requirements of pharmaceutical dosage forms.

##### 3.3.2.3 Morphological Characterization of Optimum TA-Loaded Formulations

SEM images of ideal formulations at different magnifications are presented in Figure 3. The average fiber diameter of the ideal Eudragit S100 formulation, E S 20, (259 ± 83 nm) was significantly different from that of the Eudragit RL100 formulation, E RL 32.5, (192 ± 81 nm) (*p* < 0.05). It should be noted that the diameter measurements were based on a limited number of fibers (n = 15) and therefore represent a preliminary estimation. More extensive image analysis with a larger sample size would provide a more statistically robust characterization. Several researchers have used a variety of polymers, such as poly­(ethylene oxide) and polyacrylonitrile, to examine the relationship between polymer concentration and nanofiber diameter. They have found that greater fiber diameters are the result of higher concentration and viscosity.^53,54^ Consistent with previous findings, a study using Eudragit -based nanofiber formulations for buccal application has demonstrated that increasing polymer concentration enhances solution viscosity and results in thicker, bead-free nanofibers. In addition to the concentration effect, have reported that molecular weight also plays a critical role in determining fiber diameter.^29^ Similarly, the molecular weight of Eudragit S100 was higher than that of Eudragit RL100; consequently, the fiber diameter of E S 20 was found to be higher.

**Figure 3:**
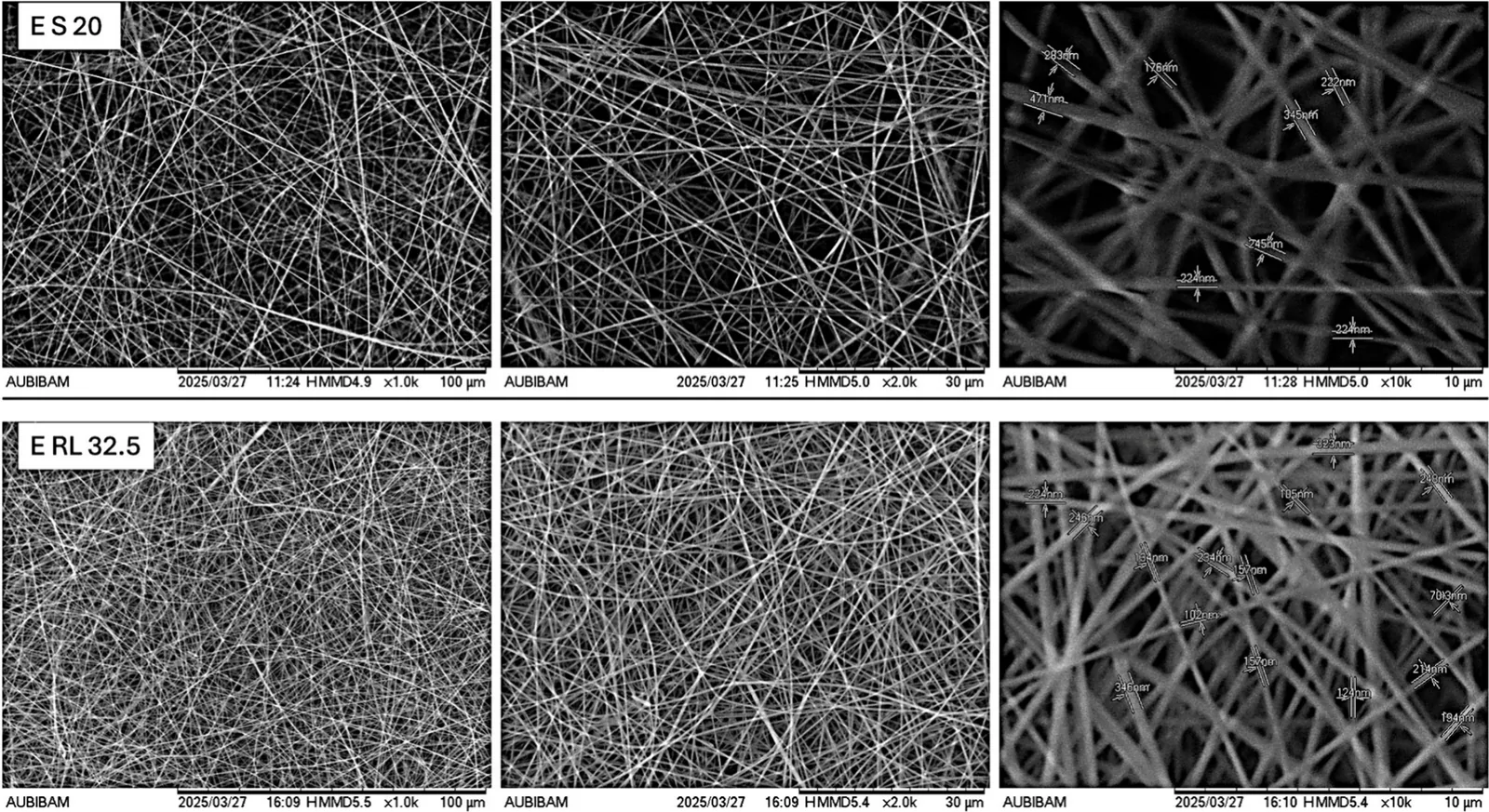
SEM images for the optimum formulations in different magnifications.

##### 3.3.2.4 Thermal Analyses

As illustrated in the DSC thermograms in Figure 4, in the DSC thermogram of TA, a large, sharp endothermic peak was observed at approximately 290 °C, which corresponds to the melting point of the active substance (TA), indicating that TA is present in a crystalline and anhydrous form. Similarly, various studies have reported an endothermic peak for TA around 290 °C.^35,55,56^

**Figure 4:**
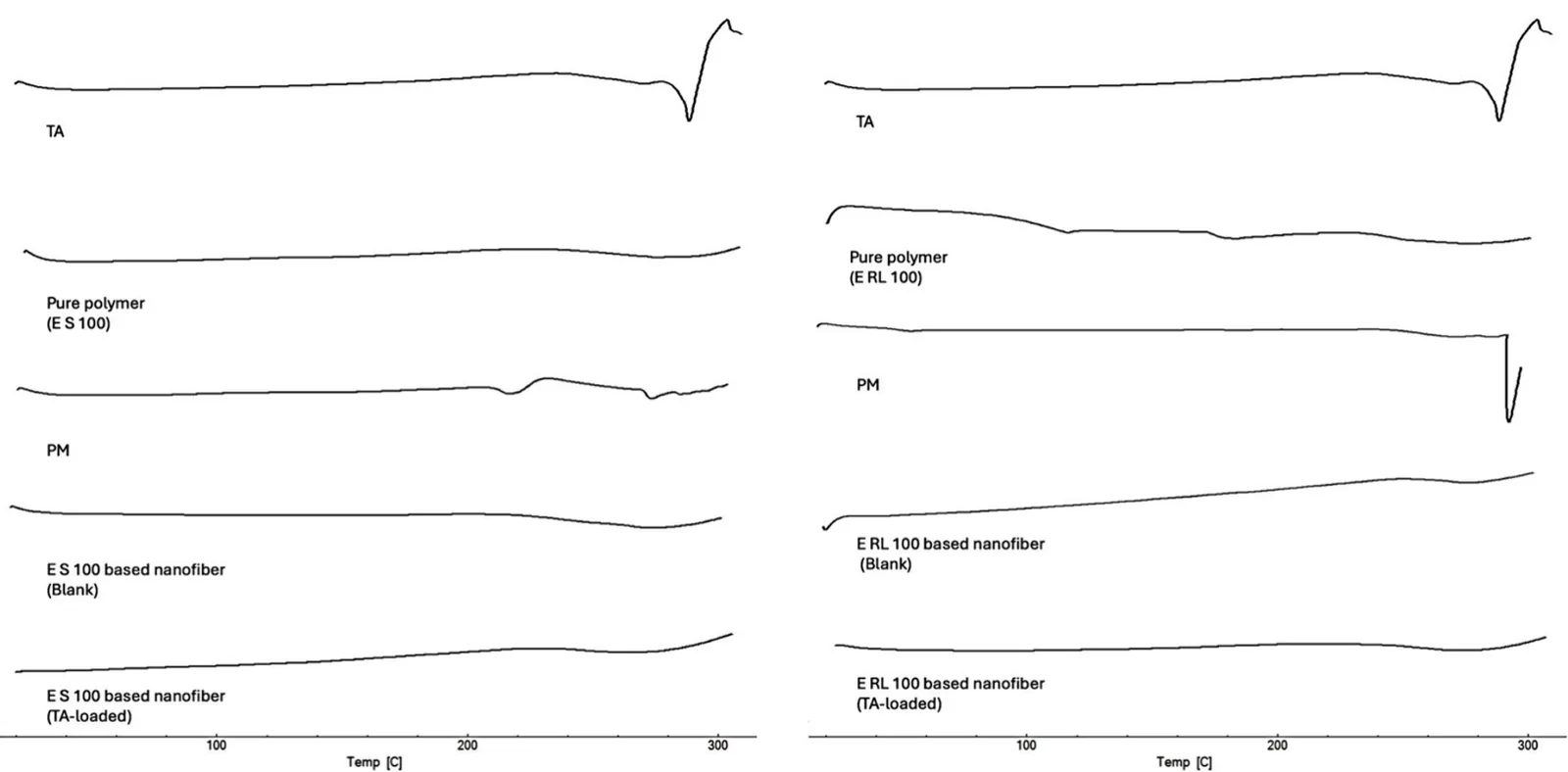
DSC thermograms of TA: Triamcinolone acetonide, E: Eudragit, PM: Physical mixture, blank, and the drug-loaded nanofibers.

As illustrated in the DSC thermograms in Figure 4, in the thermograms of the physical mixtures of TA with polymers, the intensity of the endothermic peak corresponding to TA varied depending on both the amount of TA and its interaction with the polymers. In TA-loaded nanofiber formulations, no endothermic peak related to TA was observed. This absence indicates that TA was dissolved in the selected solvents, leading to a transformation into the amorphous form, and was successfully incorporated into the polymer matrix of the nanofibers.

In line with our findings, Payab et al., (2016) also observed the absence of the characteristic TA melting peak in TA-loaded nanofibers, suggesting the transformation of TA into an amorphous state within the polymer matrix. Moreover, similar DSC interpretations have been reported in other electrospun drug-loaded nanofiber systems, supporting that nanofiber formation does not compromise the structural stability of the active compounds.^57,58^

##### 3.3.2.5 Spectroscopic Analyses

In the FTIR spectrum of TA in the Figure 5, characteristic bands were observed in the range of 3650–3200 cm^–1^ corresponding to O–H stretching vibrations, below 3000 cm^–1^ for C–H stretching vibrations, around 1700 cm^–1^ for C = O (carbonyl) stretching, between 1000–1300 cm^–1^ for C–O stretching vibrations of ether groups, and approximately at 1050 cm^–1^ for C–F stretching vibrations. The findings are consistent with previous reports.^35,55,59^

**Figure 5:**
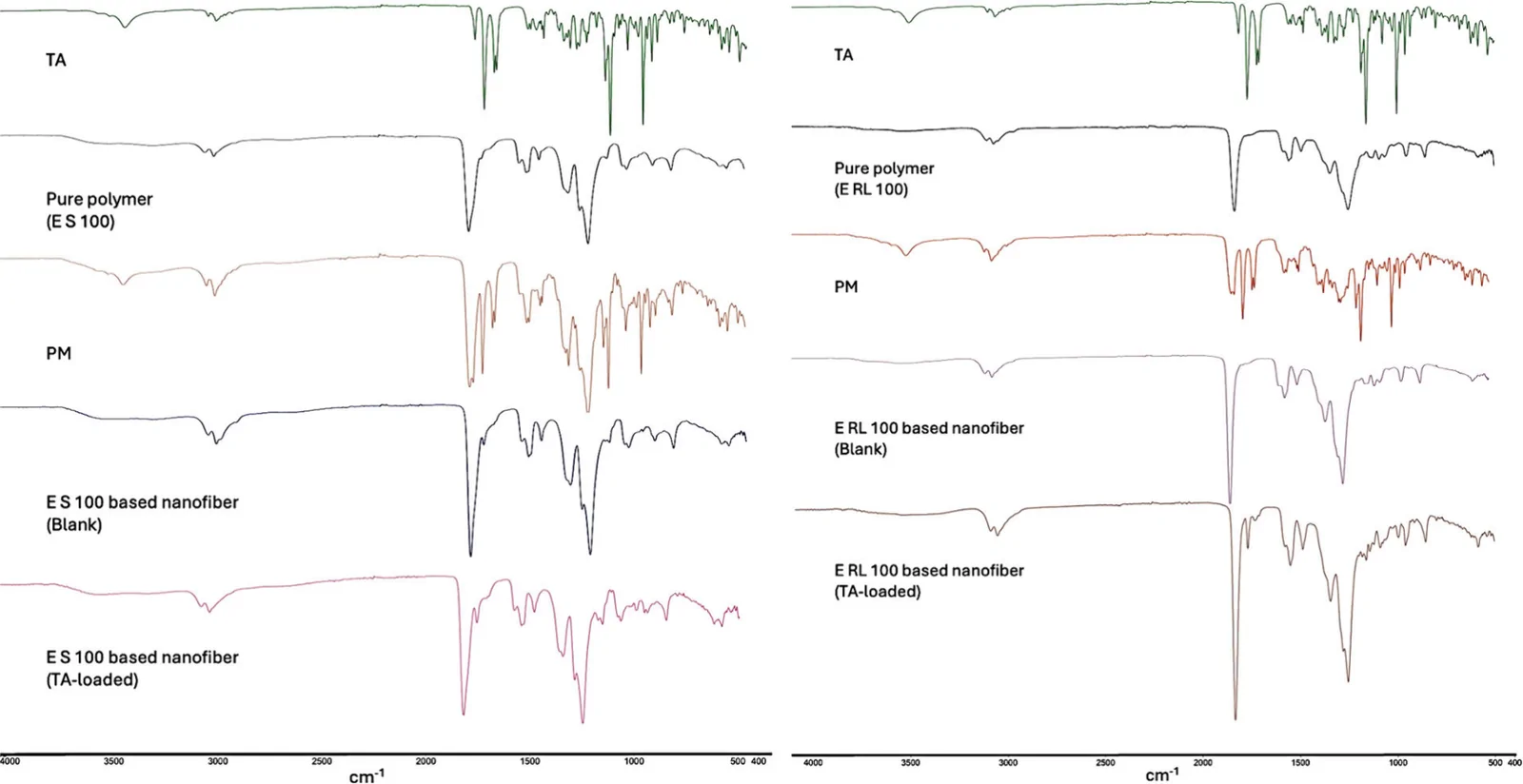
FTIR Spectra of TA: Triamcinolone acetonide, E: Eudragit, PM: Physical mixture, blank, and the drug-loaded nanofibers.

FTIR spectra were employed to evaluate the successful entrapment of the active pharmaceutical ingredient within the polymeric matrix of the nanofiber formulations. The physical mixture spectrum showed that both the main functional group bands of TA and the Eudragit polymer were preserved, and the absence of new band formation demonstrated that there was no chemical interaction between TA and the polymer, and that the interaction occurred primarily at the physical level. This suggests that TA is dispersed within the polymer matrix in the physical mixture, and its chemical structure remains unchanged. As illustrated in Figure 5, characteristic vibrational bands corresponding to TA were evident in the spectra of the physical mixtures but were absent in those of the TA-loaded nanofiber formulations. This absence strongly suggests that TA was effectively incorporated into the polymer network.

To assess the chemical stability of TA during electrospinning, comparative ^1^H NMR analyses were performed as shown in Figure 6. In the ^1^H NMR spectrum of TA in Figure 6, characteristic signals specific to TA were observed, including doublets at approximately 4.20, 4.40, 4.60, 5.00, 6.30, and 7.20 ppm, as well as a singlet around 6.10 ppm. The findings are consistent with previously reported literature data.^60^ The spectra of the TA-loaded nanofiber exhibited the distinctive signals of both the polymer and TA, indicating that the structural integrity of TA was preserved throughout the fabrication process.

**Figure 6:**
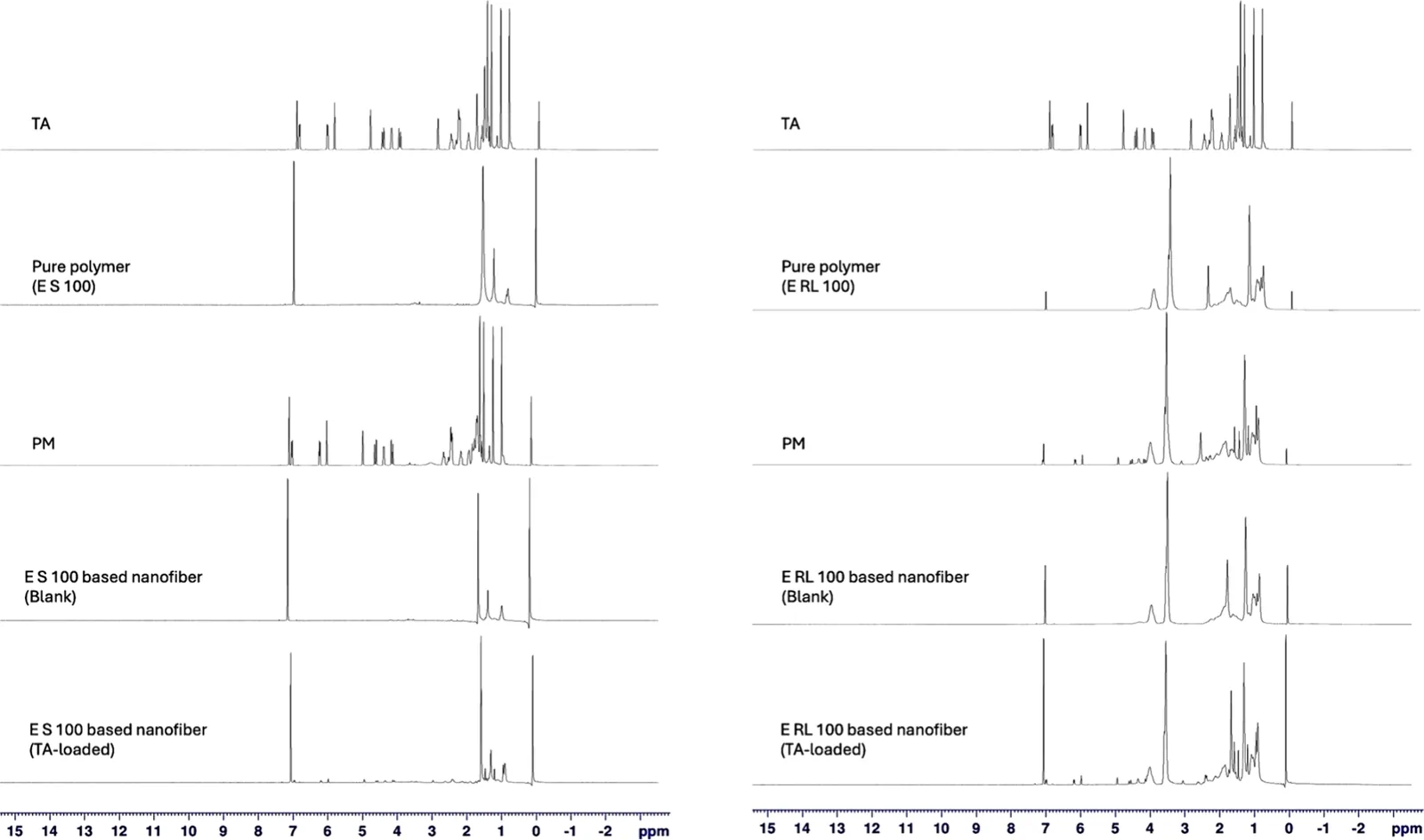
NMR Spectra of TA: Triamcinolone acetonide, E: Eudragit, PM: Physical mixture, blank, and the drug-loaded nanofibers.

### 3.4 *In Vitro* Drug Release Studies

The release studies were conducted using a Franz diffusion cell. Due to the very low aqueous solubility of TA (approximately 0.02–0.03 mg/mL in water), maintaining sink conditions in purely aqueous media is challenging.^61,62^ However, it should be noted that the presence of ethanol may influence polymer behavior and drug release kinetics and thus does not fully represent physiological buccal conditions. Although the present study focused on conventional single-fluid electrospinning as an initial formulation approach, the obtained results suggest that Eudragit-based nanofiber mats may also serve as a basis for more advanced EHDA-derived buccal delivery systems. Advanced formats such as coaxial electrospun core–sheath fibers or side-by-side/electrosprayed Janus structures may allow spatial separation of formulation components, improved drug distribution, and more precise modulation of release kinetics.^63,64^ Future studies may investigate surfactant-containing aqueous media as a more biorelevant release medium, as well as hybrid Eudragit-based electrospun or electrosprayed systems, to further improve physiological relevance, dose uniformity, residence behavior, and release control for localized buccal therapy.

As shown in Figure 7, the release profiles of the two Eudragit-based nanofiber formulations differed significantly from the earliest time points. The Eudragit S100 (E S 20) formulation consistently exhibited faster and higher cumulative release over 24 h compared to both the Eudragit RL100 (E RL 32.5) formulation and the commercial product, reaching approximately 70% cumulative release at the end of 24 h. In contrast, the release profile of the E RL 32.5 formulation was similar to the commercial product for the first 12 h, indicating comparable release performance at the initial stage. However, after this period, the E RL 32.5 formulation exhibited a more significant release, surpassing the commercial product and reaching approximately 65% cumulative release at the end of 24 h. The commercial product, however, showed the lowest release profile, remaining at approximately 50% after 24 h, exhibiting more limited drug release compared to nanofiber systems. The release of TA from electrospun Eudragit nanofibers can be explained by a combination of multiple mechanisms. Initially, the rapid release phase may be attributed to the diffusion of drug molecules located near or on the fiber surface, resulting in a burst effect, which is commonly observed in electrospun drug delivery systems.^65,66^ This is followed by a more controlled release phase governed primarily by diffusion through the polymer matrix.^65^ In addition, polymer relaxation and partial swelling may contribute to drug transport, particularly in the case of Eudragit RL100, which exhibits relatively higher permeability due to the presence of quaternary ammonium groups.^32^ For Eudragit S100, the more hydrophobic and pH-dependent nature of the polymer may limit swelling and result in a comparatively slower release profile.^33^ Furthermore, partial matrix dissolution or erosion under the experimental conditions may also contribute to drug release, especially in the presence of ethanol in the release medium.^66^ Overall, the release behavior is governed by a combination of diffusion, polymer relaxation and matrix erosion mechanisms, rather than a single dominant process, which is consistent with previous reports on electrospun polymeric drug delivery systems.^65–67^

**Figure 7:**
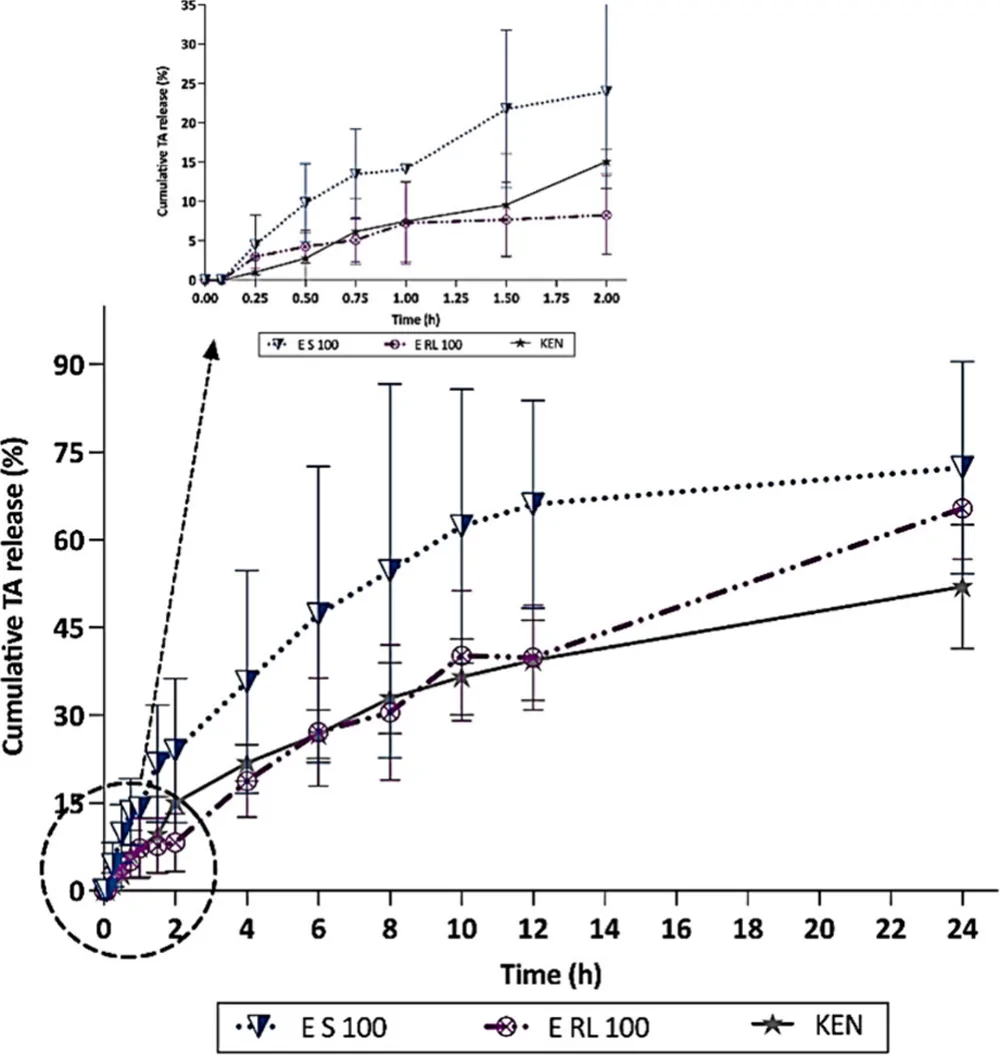
TA release profiles from the buccal nanofibers and the commercial product (*n* = 4, mean ± SD).

A study on PLGA-based TA nanofibers and nanobeads similarly exhibited prolonged and incomplete early phase release, governed by the polymer’s hydrophobicity and slow bulk erosion behavior.^68^ Another study using Eudragit RS100 matrices reported slow and sustained release extending over several hours due to the polymer’s limited permeability and insoluble structure.^35^ In a study using chitosan, chitosan-based mucoadhesive nanofibers containing TA have been shown to provide sustained release for up to 48 h due to extensive swelling and strong polymer–drug interactions.^69^ This type of prolonged release due to excessive swelling is not always optimal for rapid, intermittent local buccal applications. The Eudragit S100 and RL100 nanofibers developed in this study, by minimizing excessive swelling and exploiting polymer solubility and permeability characteristics, provide release behaviors that are better aligned with the therapeutic requirements of buccal administration. In contrast to PLGA-based systems, the nanofibrous Eudragit formulations achieved markedly faster release while avoiding the pronounced burst–plateau pattern typically observed with degradable polyesters. This distinction underscores the advantage of employing a pH-responsive polymer when a rapid therapeutic onset is required.

Release studies were evaluated using mathematical models as presented in Table 6. The commercially available preparation in ointment form was observed to have the highest r^2^ value for Higuchi kinetics. This kinetics for semisolid formulations is consistent with the literature.^70,71^ The highest correlation coefficients in nanofiber formulations were obtained with the Weibull and Korsmeyer–Peppas models. The fact that the calculated n values in the Korsmeyer–Peppas model were in the range of 0.5–1 indicates that the release mechanism is not limited to Fickian diffusion alone but rather exhibits anomalous transport characteristics where processes such as diffusion and polymer relaxation play a combined role. The fact that the β values calculated according to the Weibull model were less than 1 in all formulations supports the idea that the release initially occurs relatively rapidly and its rate decreases over time, i.e., it exhibits a diffusion-dominant release behavior.^72^

**Table 6:** Mathematical Model Fitting of TA Release Profiles from the Nanofibers and the Commercial Product

Formulation	Zero-order (R^2^)	First-order (R^2^)	Higuchi (R^2^)	Korsmeyer-Peppas n R^2^		Weibull β R^2^	
**E S 100**	0.7820	0.4411	0.9521	0.6837	0.9795	0.7409	0.9851
**E RL 100**	0.9582	0.6660	0.9825	0.7175	0.9792	0.8024	0.9792
**KEN**	0.8717	0.5477	0.9888	0.8145	0.9397	0.8864	0.9570

Similarly, the literature reports that drug release from Eudragit-based nanofibers prepared by electrospinning is not limited to a single kinetic model, and that different models may show better fit in different formulations. In a study by Reda et al., the release kinetics of nanofiber formulations were evaluated with Higuchi, Korsmeyer–Peppas, and Weibull models, and it was reported that the Weibull or Korsmeyer–Peppas models showed better fit in some formulations. It was also stated that the n values indicate an anomalous transport mechanism, meaning that release can occur not only by diffusion but also in conjunction with polymer matrix properties and relaxation processes.^29^ Additionally, it was reported that electrospun nanofibers generally exhibit a two-phase release profile, initially fast and then slower, and that this can be explained by the rapid dissolution of the drug on the fiber surface due to the high surface area, followed by continued release by diffusion through the polymer matrix.^73^ These findings are consistent with the diffusion-weighted but multimechanism release behavior obtained for nanofiber formulations in the present study.

The release behavior was discussed in light of the release profiles, polymer properties, and previous literature reports. However, the dominant release mechanism was not directly confirmed by supplementary mechanistic studies in the present work. Therefore, the proposed interpretation should be considered a mechanistic discussion rather than definitive evidence of a single release mechanism.

### 3.5 *Ex Vivo* Permeability Studies

According to the results of permeability studies conducted using the buccal mucosa (Figure 8), the highest permeability was observed with E S 100. The commercially available KEN preparation had the lowest values. After 24 h, bovine buccal tissues were examined, and the nanofibers were observed to partially maintain their integrity while adhering to the tissue, while a high amount of ointment remained on the tissue in ointment-applied tissues. For ointment-applied tissues, the percentage permeability was calculated by calculating the amount of TA transferred to the environment, the amount remaining in the tissue, and the amount of TA recovered from the residual ointment on the tissue. For nanofiber-applied tissues, the amount of TA accumulated in the tissue and retained on the tissue surface was calculated because the remaining nanofiber fragment could not be separated from the mucosa. Consequently, a high amount of the ointment was observed to remain on the tissue. This indicates that the ointment provides limited therapeutic efficacy, while nanofiber formulations can increase the effectiveness of the treatment with higher permeability.

**Figure 8:**
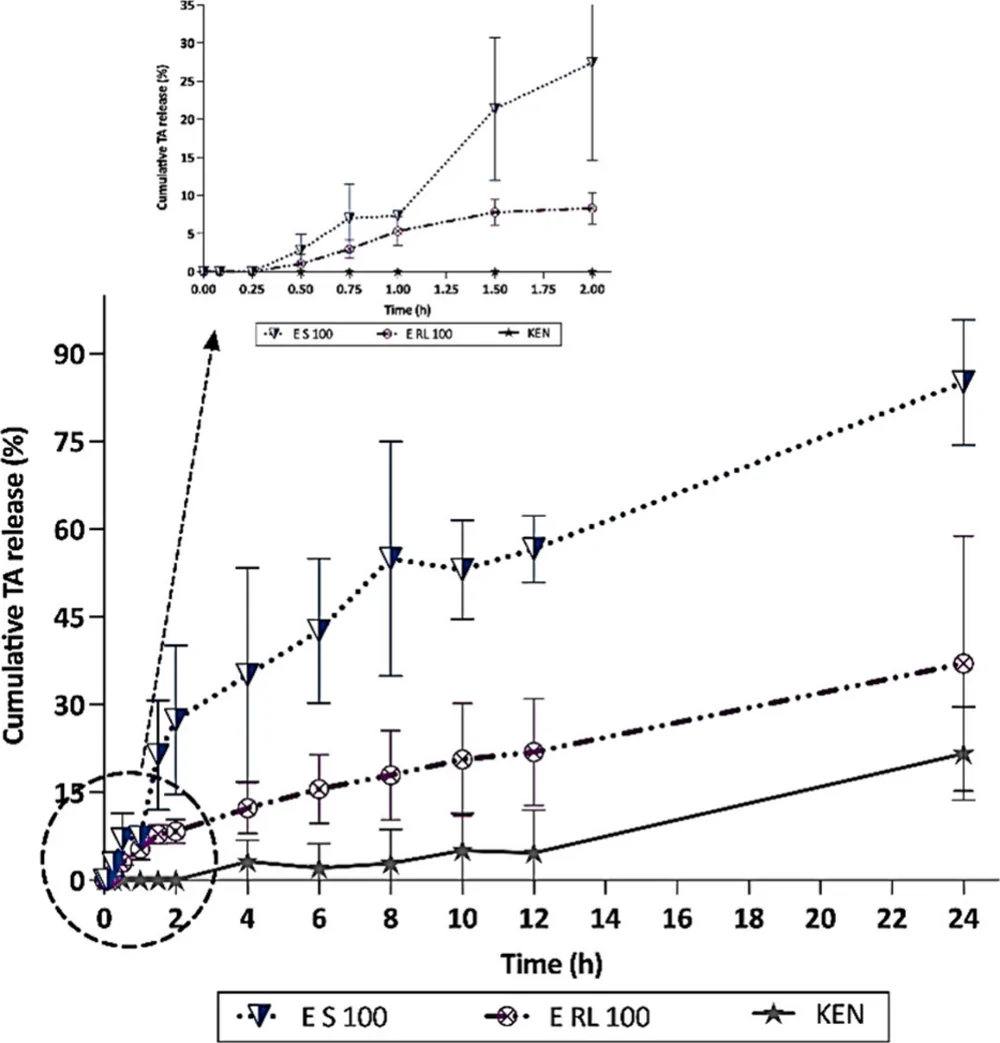
Permeation profiles of the buccal nanofibers and the commercial product (*n* = 4, mean ± SD).

Among the formulations, Eudragit S100 nanofibers exhibited the greatest permeation, followed by Eudragit RL100, while the marketed product showed the lowest permeation. Statistical analysis of the cumulative permeation values at 24 h revealed a significant difference between the E S 20 and E RL 32.5 formulations (*p* < 0.05). No significant difference was observed between E RL 32.5 and the commercial product (KEN) (*p* > 0.05), indicating comparable permeation profiles. In contrast, E S 20 showed a highly significant difference compared with the commercial formulation (*p* < 0.001), suggesting a distinct permeation behavior. The calculated Jss values were 2.494 ± 0.542 μg/cm^2^·h for E S 20, 0.882 ± 0.179 μg/cm^2^·h for E RL 32.5, and 0.269 ± 0.084 μg/cm^2^·h for the commercial formulation. Statistical comparison of the Jss values demonstrated significant differences among the formulations (*p* < 0.05). The corresponding Kp values were 0.532 cm/h, 0.127 cm/h, and 0.070 cm/h, respectively. Consistent with the cumulative permeation results, both Jss and Kp values confirmed that E S 20 exhibited the highest permeation performance, followed by E RL 32.5 and the commercial formulation. The superior permeation performance of the nanofibers can be attributed to their intimate contact with the mucosal surface, rapid hydration or partial dissolution of the polymer matrix, and efficient release of TA into the aqueous environment, in contrast to the hydrophobic base of the ointment, which retains a considerable portion of the drug.

These results are due to the permeability properties of the polymers in the formulations. Eudragit S100 is a pH-sensitive polymer; its solubility increases depending on the ambient pH, allowing for faster release of the active ingredient. In contrast, Eudragit RL100 is structurally semipermeable and suitable for controlled release; it has a sustained but slow-release pattern thanks to its quaternary ammonium groups.^74–76^ The commercially available KEN, on the other hand, is a classic semisolid formulation (ointment). Due to the oil-based structure of the ointment and the low water solubility of TA and its affinity for oil-based bases, dissolution and diffusion of TA from the matrix is more limited.^77,78^ As a result, these findings demonstrate that Eudragit-based nanofiber systems significantly improve the *ex vivo* permeation of TA compared with conventional ointment formulations, highlighting their potential for enhanced buccal delivery. The observed permeation should be interpreted in the context of local drug availability rather than systemic absorption. It should also be noted that the receptor medium composition may influence permeation behavior, and more physiologically relevant conditions could be explored in future studies.

In practical applications, the performance of buccal drug delivery systems may be influenced by physiological factors such as salivary flow, oral movements, and food intake, which can reduce residence time and potentially affect drug release and absorption, especially when unidirectional release is not controlled by a backing layer. However, buccal systems are typically designed to be administered during periods of minimal oral activity, such as between meals or before sleep, and the use of thin, flexible, mucoadhesive formulations or impermeable/semi-impermeable backing layers may improve mucosal retention and reduce dislodgement under physiological conditions. Since the nanofiber mats developed in this study did not include a backing layer, bidirectional drug release toward both the mucosa and oral cavity may occur, potentially reducing the effective local dose due to drug loss into saliva. Therefore, despite these challenges, buccal delivery remains a viable and widely explored route for localized drug delivery, particularly for the treatment of oral lesions.^30,79,80^

Although direct mucoadhesion and residence time studies were not performed, certain characteristics of the developed system suggest its potential for buccal application. The ex vivo permeation results confirmed that the formulation can effectively interact with and deliver TA across buccal tissue. In addition, Eudragit polymers, particularly RL-type copolymers, have been reported to exhibit moderate mucoadhesive properties due to electrostatic interactions between their quaternary ammonium groups and negatively charged mucin.^81,82^ Furthermore, electrospun nanofibers possess high surface area and flexibility, which may enhance intimate contact with the mucosal surface and contribute to improved retention.^11^ However, it should be noted that these assumptions require direct experimental validation, and future studies should include quantitative mucoadhesion and residence time measurements.

## 4 Conclusions

In this study, TA-loaded nanofiber formulations based on two different Eudragit polymers were successfully developed as an alternative to the commercially available buccal ointment used in the treatment of oral ulcers. Bead-free electrospun nanofiber mats were obtained and comprehensively characterized, confirming the successful incorporation of TA into the polymer matrices with diameters in the nanometer range. As part of process optimization, the effect of the collector plate movement stroke on the mechanical properties of the nanofibers was systematically investigated, revealing a significant influence on their brittleness and durability while maintaining constant electrospinning parameters for fair comparison. *In vitro* release and *ex vivo* permeation studies demonstrated that the developed nanofiber formulations exhibited enhanced drug release and higher mucosal permeation compared with the commercial ointment. The present work contributes to the growing field of electrospun drug delivery systems by demonstrating the feasibility of using Eudragit-based nanofibers for the delivery of poorly water-soluble corticosteroids. Compared to conventional dosage forms such as ointments or gels, electrospun nanofibers offer advantages including high surface area, rapid wetting, and tunable drug release profiles. These findings highlight the potential of electrospinning as a versatile platform for designing advanced buccal drug delivery systems.^16,83^ Although *ex vivo* permeation studies demonstrated that TA can effectively penetrate the buccal mucosa, the lack of direct mucoadhesion and residence time evaluation limits the ability to fully predict *in vivo* performance under dynamic oral conditions. The differences in release and permeation behavior between Eudragit S100- and Eudragit RL100-based nanofibers reflect the distinct permeability and physicochemical properties of these polymers, emphasizing the importance of polymer selection in buccal nanofiber design. As a result, these findings indicate that Eudragit-based electrospun nanofibers represent a promising alternative platform for localized buccal delivery of TA. Overall, this work represents one of the first studies investigating TA-loaded nanofibers in an *ex vivo* buccal model and highlights their potential for buccal corticosteroid delivery. However, the present findings are limited to *in vitro* and *ex vivo* conditions. Future studies should therefore focus on assessing mucoadhesive properties, retention behavior, and formulation stability in the buccal environment, *in vivo* investigations, and safety assessments to confirm the clinical potential of the developed nanofiber systems. In addition, more physiologically relevant release conditions may be considered to better simulate the oral cavity. Overall, the developed system represents a promising platform for localized buccal drug delivery, warranting further investigation.
